# Phosphorylation of *Arabidopsis* SINA2 by CDKG1 affects its ubiquitin ligase activity

**DOI:** 10.1186/s12870-018-1364-8

**Published:** 2018-07-16

**Authors:** Yang Chen, Mohamed Fokar, Miyoung Kang, Naichong Chen, Randy D. Allen, Yaofeng Chen

**Affiliations:** 1 College of Agriculture, Northwest Agriculture and Forestry University, Yangling, Shaanxi 712100 Republic of China; 2 Institute for Agricultural Biosciences, Oklahoma State University, Ardmore, OK 73401 USA

**Keywords:** CDKG1, SINA2, Phosphorylation, Ubiquitin ligase activity, Stress response

## Abstract

**Background:**

SEVEN IN ABSENTIA (SINA) is a RING domain-containing ubiquitin ligase involved in *Drosophila* eye formation. SINA-like proteins in plants are involved in several signaling pathways. Of the 18 SINA-like proteins identified in Arabidopsis, SEVEN IN ABSENTIA 2 (SINA2) lacks a canonical RING domain and is thought to lack ubiquitin ligase activity.

**Results:**

Our results show that SINA2 has E3 ligase activity in vitro, raising the possibility that a modified B-box domain may compensate for its lack of a RING domain. SINA2 physically interacts with the nuclear protein CYCLIN-DEPENDENT KINASE G1 (CDKG1), which acts as a positive regulator of plant responses to abiotic stress. CDKG1 is expressed in multiple tissues and its expression increased in response to abscisic acid (ABA) and osmotic stress. Transgenic Arabidopsis plants that ectopically express CDKG1 exhibit increased tolerance to ABA and osmotic stress treatments during seed germination and cotyledon development, while the loss-of-function *cdkg1* mutant plants show reduced tolerance to ABA and osmotic stress treatments. Moreover, CDKG1-dependent phosphorylation of SINA2 positively affects its E3 ubiquitin ligase activity.

**Conclusions:**

Based on these results, we propose that CDKG1 modulates SINA2 ubiquitin ligase activity to regulate its effect on plant responses to ABA and osmotic stress.

**Electronic supplementary material:**

The online version of this article (10.1186/s12870-018-1364-8) contains supplementary material, which is available to authorized users.

## Background

Unlike animals, plants are sessile and are unable to avoid stressful environmental conditions that often limit their growth and development. To survive these stresses, plants have evolved complex biochemical and physiological strategies that are controlled by a network of signaling events. Post-translational modifications, such as phosphorylation and ubiquitination, play crucial roles in regulating many biological processes. Phosphorylation results in activation or deactivation of many enzymes and receptors by inducing conformational changes in protein structure [10, 13], while, in many cases, ubiquitination can affect protein quality and stability by attaching ubiquitin chains to target proteins, which can then be degraded by 26S proteasomes [36, 45].

The specificity of ubiquitination is determined largely by E3 ubiquitin ligases, which are capable of recognition, recruitment, and transfer of ubiquitin from an E2 ubiquitin conjugating enzyme intermediate to the target protein [14, 25, 40, 45]. More than 1400 genes that encode putative E3 ubiquitin ligases have been identified in the Arabidopsis genome [45]. These E3 ubiquitin ligases can be divided into two types according to the number of subunits. The single-subunit enzymes include RING, U-box, and HECT domain-containing enzymes, while multiple-subunit CULLIN-based E3 ligase complexes include the Skp1-CULLIN1-F-box complex, CUL3-BTB and CUL4-DDB1 [37].

Seven in absentia (SINA) was first identified in *Drosophila melanogaster* where it is necessary for the development of R7 photoreceptor cells in the eye. This is accomplished by ubiquitination of the transcriptional repressor Tramtrack, targeting it for proteasomal degradation [3]. A single gene with extensive sequence homology to *Drosophila SINA*, named *Siah*, was identified in mouse [6] and Human *SIAH1* and *SIAH2* are also closely related to *Drosophila SINA* [15]. Unlike animals, plants contain large families of highly conserved seven in absentia homologs. For example, eighteen members of a *SINA-like* gene family were identified in Arabidopsis [34]. In most cases, the E3 ubiquitin ligase activity of SINAs is dependent on the N-terminal Really Interesting New Gene (RING) finger domain.

The role of SINAs as E3 ubiquitin ligases in signaling pathways has been investigated in plants. For example, in rice, OsDIS1 was shown to act as a negative regulator of drought stress responses, possibly by affecting the stability of OsNek6 [29]. The tomato ubiquitin ligase SINA3 plays a negative role in plant responses to *Pseudomonas* infection by promoting the degradation of the defense-related transcription factor NAC1 via poly-ubiquitination [24]. In Arabidopsis, SINAT5 is an E3 ubiquitin ligase that attenuates auxin signaling in roots by targeting the transcription factor NAC1 for degradation [47] and is also involved in regulation of flowering time through posttranslational regulation of FLC and LHY [31, 32]. In addition, the interaction between the glycolytic enzyme GAPC1 and the E3 ubiquitin ligase SINAL7 enhances the effectiveness of GAPC1, increasing the glycolytic flux within cells. This interaction is dependent on the Lys231 residue of GAPC1; however, the glycolytic activity of GAPC1 is completely abolished when SINAL7 catalyzes its mono-ubiquitination at residue Lys76 [35]. Arabidopsis SINA2, which lacks a conserved N-terminal RING domain (see Additional file 1: Figure S1) and was predicted not to have E3 ubiquitin ligase activity, functions as a positive regulator of the ABA-dependent stress signaling pathway [1] but how SINA2 affects ABA-mediated responses to stress is unknown.

The activity of certain E3 ubiquitin ligases has been shown to be regulated by phosphorylation. In some cases, phosphorylation of an E3 ubiquitin ligase affects its ability to interact with specific E2 enzymes or substrates. For example, the phosphorylation of Parkin at Ser131 by Dyrk1A negatively regulates its E3 ubiquitin ligase activity by reducing its binding affinity to the E2 enzyme UbcH8 and substrate TRAF2 [17]. Tyrosine phosphorylation of the E3 ubiquitin ligase TRIM21 at Y393 is required, not only to maintain its activity, but also to promote the interaction between TRIM21 and its substrate IRF3 [42]. In addition, E3 ubiquitin ligase activity can be modulated by phosphorylation through changes in state of the E3 ligase itself. The non-phosphorylated form of the HECT E3 ligase Itch does not have ubiquitin ligase activity due to an intramolecular interaction between its HECT and WW domains. JUK1-mediated phosphorylation of S199, S232, and T222 disrupts this self-inhibitory interaction by forcing the WW domain to adopt a different conformation, greatly enhancing the catalytic activity of Itch [11]. Tyrosine phosphorylation of c-Cbl also removes the inhibitory effects of the TKB domain, resulting in activation of its E3 ubiquitin ligase activity [20]. Moreover, the phosphorylation of Y363 induces the activation of Cbl-b by removing the interdomain interaction between RING and TKB-H and exposing the E2 binding surface of the RING domain [22].

In this work, we show that SINA2, which lacks a canonical RING domain, retains auto-ubiquitination activity in vitro. SINA2 also physically interacts with CYCLIN-DEPENDENT KINASE G1 (CDKG1), a protein kinase that acts as a positive regulator of plant response to ABA-related abiotic stress. SINA2 is phosphorylated by CDKG1 on Ser/Thr sites and de-phosphorylation of Ser/Thr/Tyr results in the loss of ubiquitin ligase activity. Our results indicate that up-regulation of the ubiquitin ligase activity of SINA2 by CDKG1-dependent phosphorylation, could lead to altered stress responses in Arabidopsis.

## Results

### *sina2*^*−*^ mutant seedlings are hypersensitive to ABA and abiotic stress

**Fig. 1:**
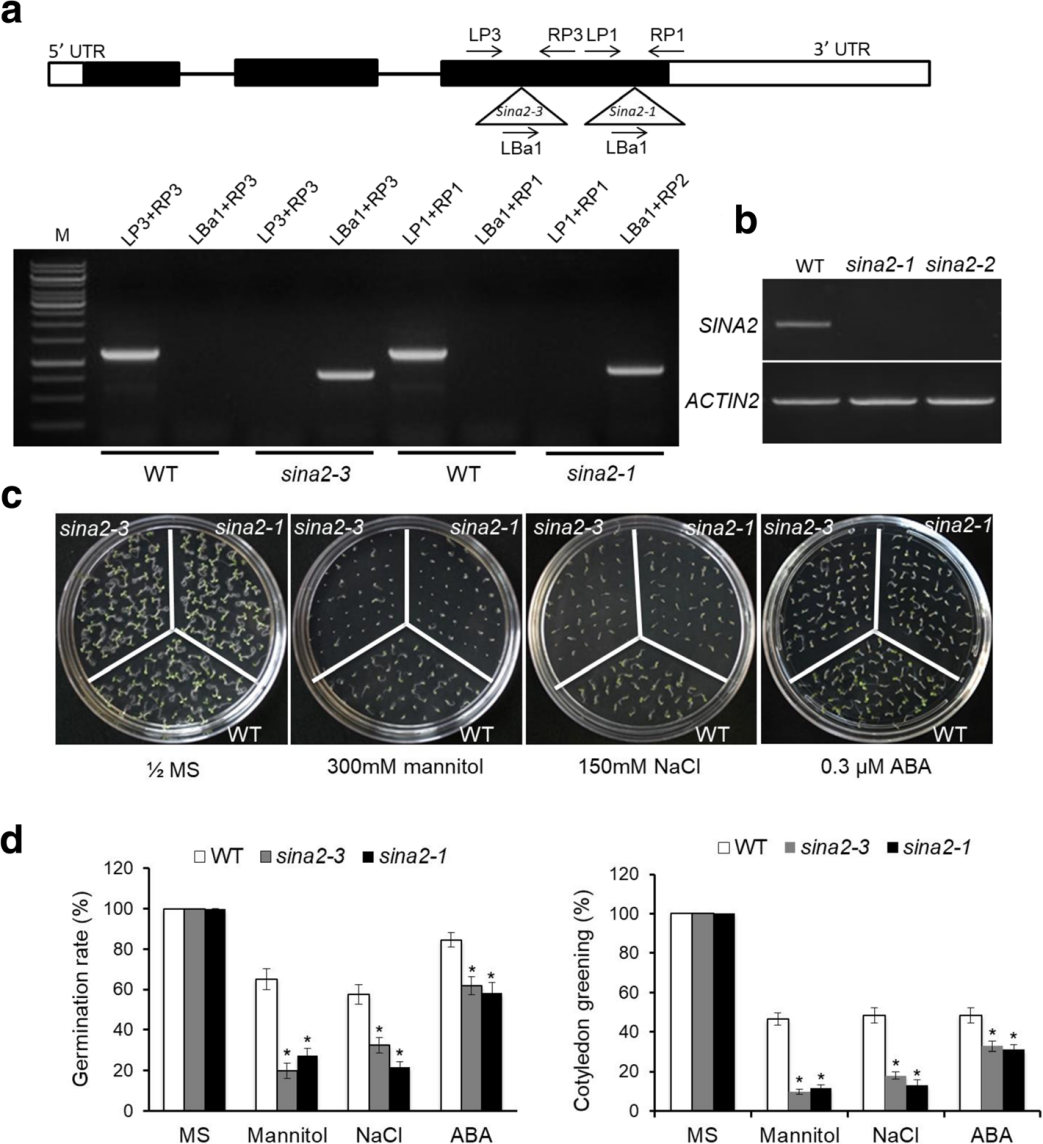
Molecular characterization of *sina2* mutants and comparative analysis of their sensitivity to ABA and abiotic stress. **a** Schematic diagram of T-DNA insertions and PCR-validation of *sina2–3* and *sina2–1* mutants (primer sequences are shown in Additional file 1 Table S1). Boxes and lines denote exons and introns, respectively. The positions of the T-DNA inserts are indicated by triangles and primer positions are indicated by arrows. **b** RT-PCR analysis of *SINA2* transcript expression in wild type (WT), *sina2–3,* and *sina2–1* lines. *AtACTIN2* was used as an internal standard. **c** Phenotype analysis of *sina2* mutant seeds sown on growth media without amendment or containing 300 mM mannitol, 150 mM NaCl, or 0.3 μM ABA. **d** Quantitative analysis of germination and post-germinative cotyledon greening on 300 mM mannitol, 150 mM NaCl, or 0.3 μM ABA. The percentage of germinated seeds was scored 4 days after stratification and cotyledon greening data was collected 7 days after germination. Each data point represents a mean of 60 seeds ± SD. Asterisks indicate significant differences from WT by student’s *t*-test (*P* < 0.01)

SINA2 was reported to positively regulate drought stress responses in mature Arabidopsis plants in an ABA dependent manner [1]; however, these authors could not detect significant differences in the germination and early seedling development between *sina2* T-DNA knockout lines, *SINA2* over-expressing transgenic lines and WT. To better understand the role of SINA2 in seedlings, we tested rates of seed germination and cotyledon greening of the same Arabidopsis *sina2* T-DNA insertion mutants in response to salt and osmotic stress treatments, as well as ABA. Two independent T-DNA insertion lines, SALK_099866 (named *sina2–3*) and SALK_129594 (named *sina2–1*), were obtained from the ABRC seed stock center. It should be noted that the SALK_099866 T-DNA insertion is associated with two loci in the TAIR database. Flanking sequence of the T-DNA insert match coding sequences in the third exon of the *SINA2* gene (AT3G13672), along with sequences annotated as the 3′ untranslated region of the adjacent gene (AT3G13670). Homozygous mutant lines were verified by diagnostic PCR using *SINA2*-specific and T-DNA border-specific primers (Fig. 1a), and their mRNA-null phenotype was confirmed by RT-PCR analyses using *SINA2*-specific primers (Fig. 1b). When grown on half-strength MS medium, no obvious morphological or developmental differences were apparent between wild-type (WT) and either of the *sina2* mutant lines (Fig. 1c). However, in the presence of 0.3 μM ABA, germination of *sina2* seeds was delayed relative to WT, with only 58.2% of *sina2–1* and 61.8% of *sina2–3* seeds germinated by 4 days after stratification, while 84.5% of WT seeds germinated under the same conditions (Fig. 1d). At 7 days after stratification, the percentage of seedlings with expanded green cotyledons was substantially lower for both *sina2* mutant lines than for WT under ABA treatment. Exposure to osmotic or salinity stress caused by incorporating either 300 mM mannitol or 150 mM NaCl into the media, also reduced the rates of germination and cotyledon greening more severely in both *sina2* mutant lines than in WT. These results indicate that SINA2 also helps to promote seed germination and early seedling development under osmotic and salinity stress conditions, possibly by reducing their sensitivity to ABA.

### SINA2 is a functional E3 ubiquitin ligase

**Fig. 2:**
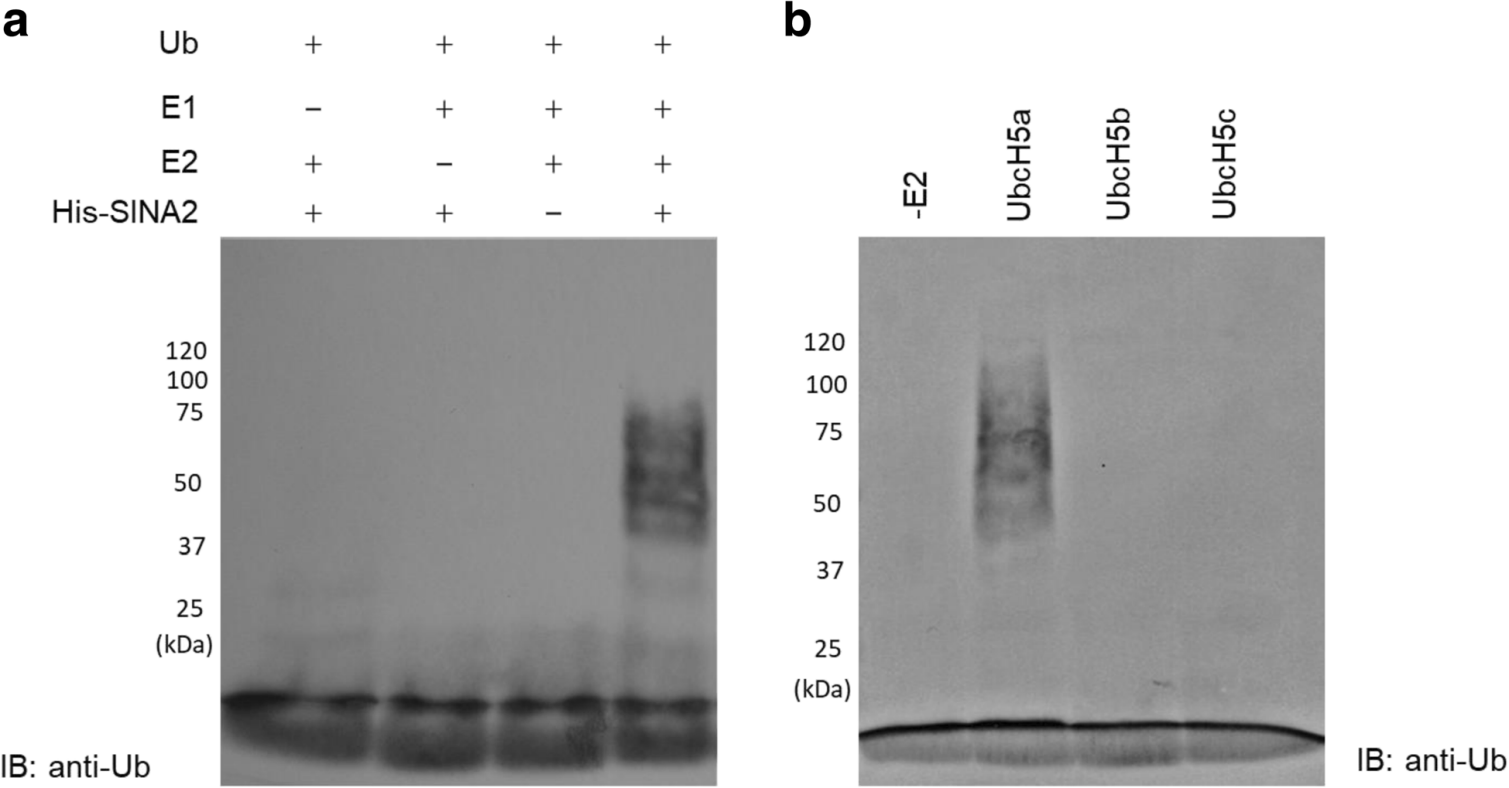
E3 ubiquitin ligase activity of SINA2. **a** In vitro auto-ubiquitination activity assays were performed in the presence of human E1, human E2 (UbcH5a) and ubiquitin. Samples were resolved by SDS-PAGE on 10% gels and detected by immunoblot analysis using anti-Ub. **b** Various human E2 enzymes were tested for ubiquitin-conjugating activity in the presence of His-SINA2 and only assays containing UbcH5a showed positive results

Since the amino terminus of AtSINA2 is truncated relative to other AtSINAs, it does not contain the canonical RING finger domain thought to be required for E3 ubiquitin ligase activity (see Additional file 1: Figure S1). Therefore, SINA2 was predicted to lack this activity [1]. To test this prediction, recombinant SINA2 was expressed in *E. coli* as a fusion protein with a 6 × His epitope tag and the tagged protein was purified from the soluble protein fraction using Ni affinity chromatography. In the presence of ubiquitin, human E1 and human E2 (UBCH5a), His-SINA2 catalyzed the formation of high molecular weight auto-ubiquitin chains (Fig. 2a), while no ubiquitinated products were detected in reactions that lacked E1, E2, or His-SINA2. These results indicate that, in spite of lacking a RING domain, SINA2 has E3 ubiquitin ligase activity in vitro. Since E3 ubiquitin ligases catalyze the transfer of ubiquitin from specific E2 ubiquitin conjugating enzymes to substrates, we tested three different E2 enzymes namely, UBCH5a, UBCH5b, and UBCH5c, for their ability to support the E3 ubiquitin ligase activity of SINA2 in vitro. As shown in Fig. 2b, SINA2 auto-ubiquitination was only detected in reactions that included UBCH5a.

### SINA2 interacts with CDKG1

**Fig. 3:**
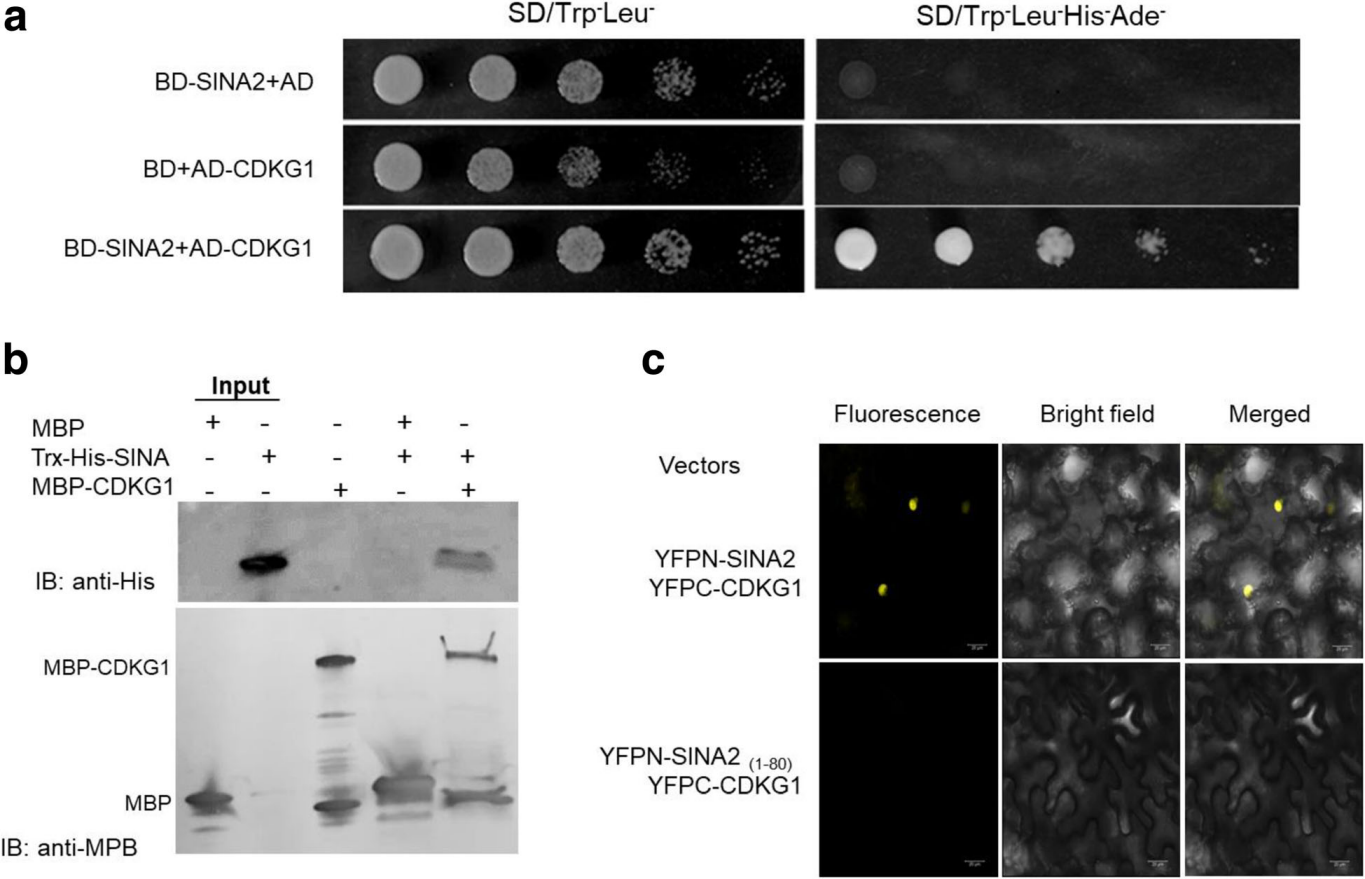
Interaction between CDKG1 and SINA2. **a** To test interactions by yeast two-hybrid assays, vectors were co-transformed into yeast AH109 strain in different combinations: 1) pGBKT7-SINA2 and pGADT7; 2) pGBKT7 and pGADT7-CDKG1; 3) pGBKT7-SINA2 and pGADT7-CDKG1. Transformants were inoculated onto SD Trp^−^Leu^−^medium and replica plated onto SD Trp^−^Leu^−^His^−^Ade^−^ medium and incubated for 4 days. **b** In vitro pull-down assay. MBP-CDKG1 was used to pull down 6 × His labeled SINA2. The MBP tag alone was used as the negative control. Immobilized MBP and MBP-CDKG1 were detected by Coomassie brilliant blue staining. **c** In vivo BiFC assay. The plasmids YFPN-SINA2 and YFPC-CDKG1 were co-transformed by agroinfiltration into *N. benthamiana* leaves. Images were captured after 3 days using a confocal laser-scanning microscopy. Strong luminescence was localized in nuclei of epidermal cells. This signal was lost when SINA2_(1–80)_ was used in place of intact SINA2

Yeast two-hybrid screening was performed to identify potential protein partners that interact with SINA2. A cDNA sequence that encodes CYCLIN-DEPENDENT KINASE G1 (CDKG1, At5G63370.1), was found to restore growth of yeast cells on high stringency selection media. The CDKG1 coding sequence was cloned into the pGADT7 vector and co-transformed with pGBKT7-SINA2 into yeast, resulting in yeast growth on SD/Trp^−^Leu^−^Ade^−^His^−^ medium. No yeast growth was observed with the other combinations, pGBKT7 + pGADT7-CDKG1 and pGBKT7-SINA2 + pGADT7 (Fig. 3a). Reciprocal yeast 2-hybrid assays showed identical results (see Additional file 1: Figure S2a). To determine which part of SINA2 is responsible for its interaction with CDKG1 in yeast, we generated two SINA2 deletion constructs, one that encodes the amino terminal portion of SINA2 from amino acids 1 to 80 (BD-SINA2_(1–80)_) and a second construct that encodes the C-terminal portion of SINA2 from amino acids 77 to 216 (BD-SINA_(77–216)_) and tested them for interactions with CDKG1 in yeast 2-hybirid assays (see Additional file 1: Figure S2b). The results showed that the 80 amino acids at the N-terminal of SINA2, including the putative B-box domain (see below), are not capable of interacting with CDKG1 and are not required for the interaction of SINA2 with CDKG1. Next, the interaction between SINA2 and CDKG1 was confirmed by in vitro pull-down assays. Two recombinant proteins, His-SINA2 and MBP-CDKG1, were expressed in *E. coli* and purified. After co-incubation of these proteins, immunoselection using anti MBP and immunoblot analysis using anti-HIS showed that MBP-CDKG1 was able to pull down His-SINA2. His-SINA2 pulldown was not detected in control assays that contained MBP rather than MBP-CDKG1 (Fig. 3b).

### Characterization and localization of CDKG1

The transcribed sequence of *AtCDKG1* includes no introns and encodes a 612-amino-acid polypeptide with a putative molecular weight of 69.62 kDa and a predicted isoelectric point of 6.11. Multiple sequence alignment showed that the derived AtCDKG1 amino acid sequence has over 60% identity with CDKG1 homologs from various plant species and includes a highly conserved PK domain (see Additional file 1: Figure S3a). AtCDKG1 is 99% identical to its homolog from *Arabidopsis lyrata* (AlCDKG1) and also shares 53.3% identity with AtCDKG2 (see Additional file 1: Figure S3b).

Subcellular localization of SINA2 and CDKG1 was analyzed by transient expression of GFP-SINA2 and GFP-CDKG1 fusion proteins in *Nicotiana benthamiana* leaf epidermal cells using agroinfiltration. GFP signals were detected using scanning confocal microscopy 48 h after infiltration. As reported by *Bao* et al. [1] GFP-SINA2 was detected in both cytosolic and nuclear cellular compartments in a pattern similar to GFP alone, while GFP-CDKG1 was located exclusively in nuclei (see Additional file 1: Figure S4). Interaction of SINA2 and CDKG1 *in planta* was confirmed by bimolecular fluorescence complementation (BiFC) assays. Vectors that express YFPN-SINA2 and YFPC-CDKG1 were co-transformed into *N. benthamiana* leaf epidermal cells by agroinfiltration. Strong fluorescence signals were observed in nuclei of cells that transiently co-express YFPN-SINA2 and YFPC-CDKG1 (Fig. 3c). However, when YFPC-CDKG1 was co-expressed with SINA2 lacking the C-terminus (YFPN-SINA2_(1–80)_), no fluorescence signal was detected. These results demonstrate that SINA2 interacts with CDKG1 in nuclei of plant cells.

### Expression of *CDKG1*

**Fig. 4:**
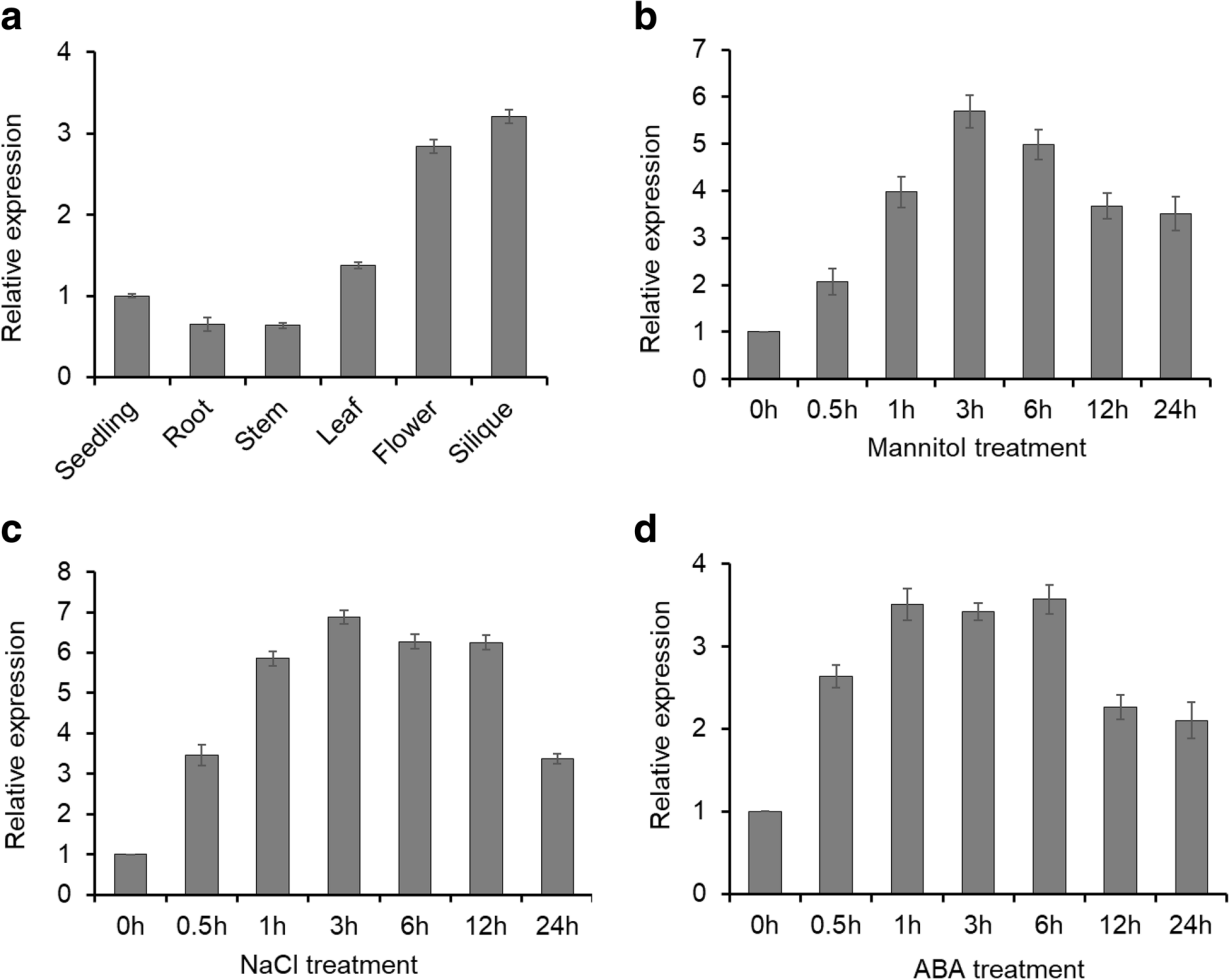
Expression pattern of *CDKG1*. **a** Relative expression of the *CDKG1* gene in different tissues of mature Arabidopsis plants assayed using reverse transcriptase quantitative PCR (RT-qPCR). **b** RT-qPCR analyses of CDKG1 expression in two week old Arabidopsis seedlings in response to 200 mM mannitol,(**c**) 200 mM NaCl, or (**d**) 100 μM ABA. Seedling were grown on MS media and transferred to fresh plates containing MS media with the supplements shown and samples were collected at 0 (control), 0.5, 1, 3, 6, 12, 24 h after the initiation of treatment. The relative expression was normalized using *ACTIN2*. Data represent the average of three independent experiments ± SD with three technical repeats each

Quantitative RT-PCR analysis indicated that *CDKG1* is ubiquitously expressed in seedlings and in all organs examined, including roots, stems, leaves, flowers and siliques of Arabidopsis plants (Fig. 4a). Highest expression of *CDKG1* mRNA was detected in flowers and siliques of mature plants. To explore the possible roles of CDKG1 in plant responses to abiotic stress, we performed RT-qPCR to compare of *CDKG1* transcript levels in plants exposed to different stress regimes. Increased *CDKG1* expression was detected within 0.5 h of treatment with 300 mM mannitol and a 6-fold increase, relative to untreated plants, was seen within 3 h, followed by a gradual decline (Fig. 4b). Similar to mannitol treatment, *CDKG1* expression was rapidly induced in response to salinity stress (150 mM NaCl), with a maximal increase of about 7-fold at 3 h (Fig. 4c). In response to ABA treatment, *CDKG1* expression was induced by more than 3-fold after 1 h and expression remained at this level through 6 h before declining gradually (Fig. 4d). These results indicate that *AtCDKG1* expression is responsive to ABA and abiotic stress treatments and indicates that this gene may be involved in the abiotic stress response pathway in Arabidopsis.

### *CDKG1* is involved in plant responses to ABA and ABA-related stress

**Fig. 5:**
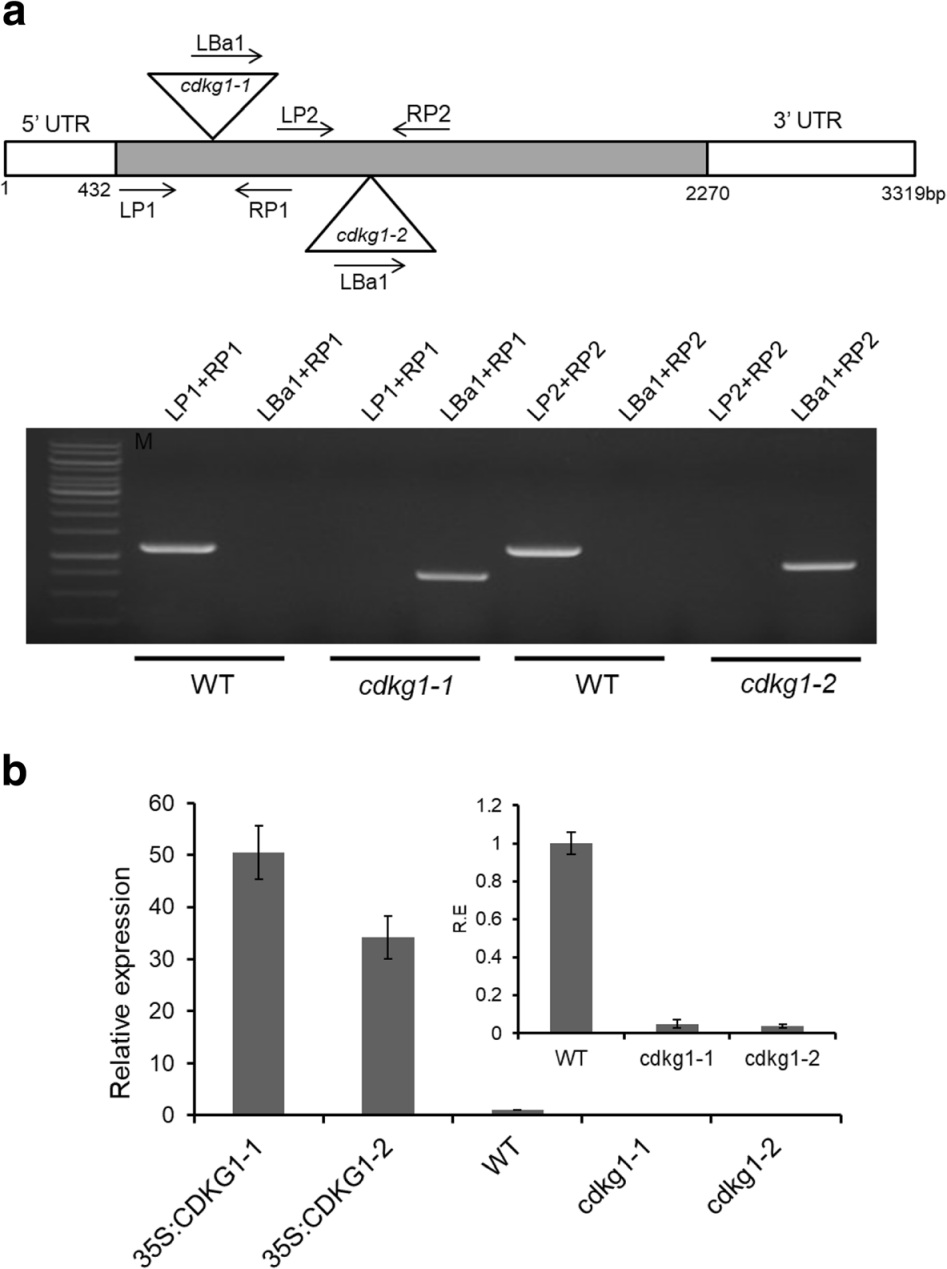
Molecular characterization of *35S:CDKG1* and *cdkg1* plants. **a** Schematic diagram of T-DNA insertion in *cdkg1–1* and *cdkg1–2* mutants and PCR validation of *cdkg1–1* and *cdkg1–2* mutants (see Additional file 1 Table S1 for primer sequences). Box denotes the single exon of the *CDKG1* gene. The positions of the T-DNA inserts are indicated by the triangles and the qPCR primers sites are indicated by the arrows. **b** RT-qPCR analysis of *CDKG1* transcript expression in *cdkg1*, WT and *35S:CDKG1* lines. *AtACTIN2* was used as an internal standard. Data represent the average of three independent assays ± SD with three technical repeats each

The role of *CDKG1* in plant responses to ABA and ABA-related stress was investigated using a reverse genetic approach. Transgenic Arabidopsis plants that constitutively express *CDKG1* under control of the cauliflower mosaic virus (CaMV) 35S promoter were generated. In addition, two independent *cdkg1* knockout mutant lines that had unique T-DNA insertion sites within the *CDKG1* exon were obtained and their structures were confirmed by genomic PCR analysis (Fig. 5a). The expression of *CDKG1* mRNA in transgenic *35S:CDKG1* lines and *cdkg1* knockout mutants was verified by RT-qPCR analysis (Fig. 5b). Two independent *35S:CDKG1* transgenic lines showed substantially higher levels of *CDKG1* transcripts than WT (between 30 and 50-fold), while neither of the *cdkg1* mutant lines produced detectable signals for *CDKG1* transcripts under the conditions used, indicating that they are *cdkg1*^*null*^ alleles.

**Fig. 6:**
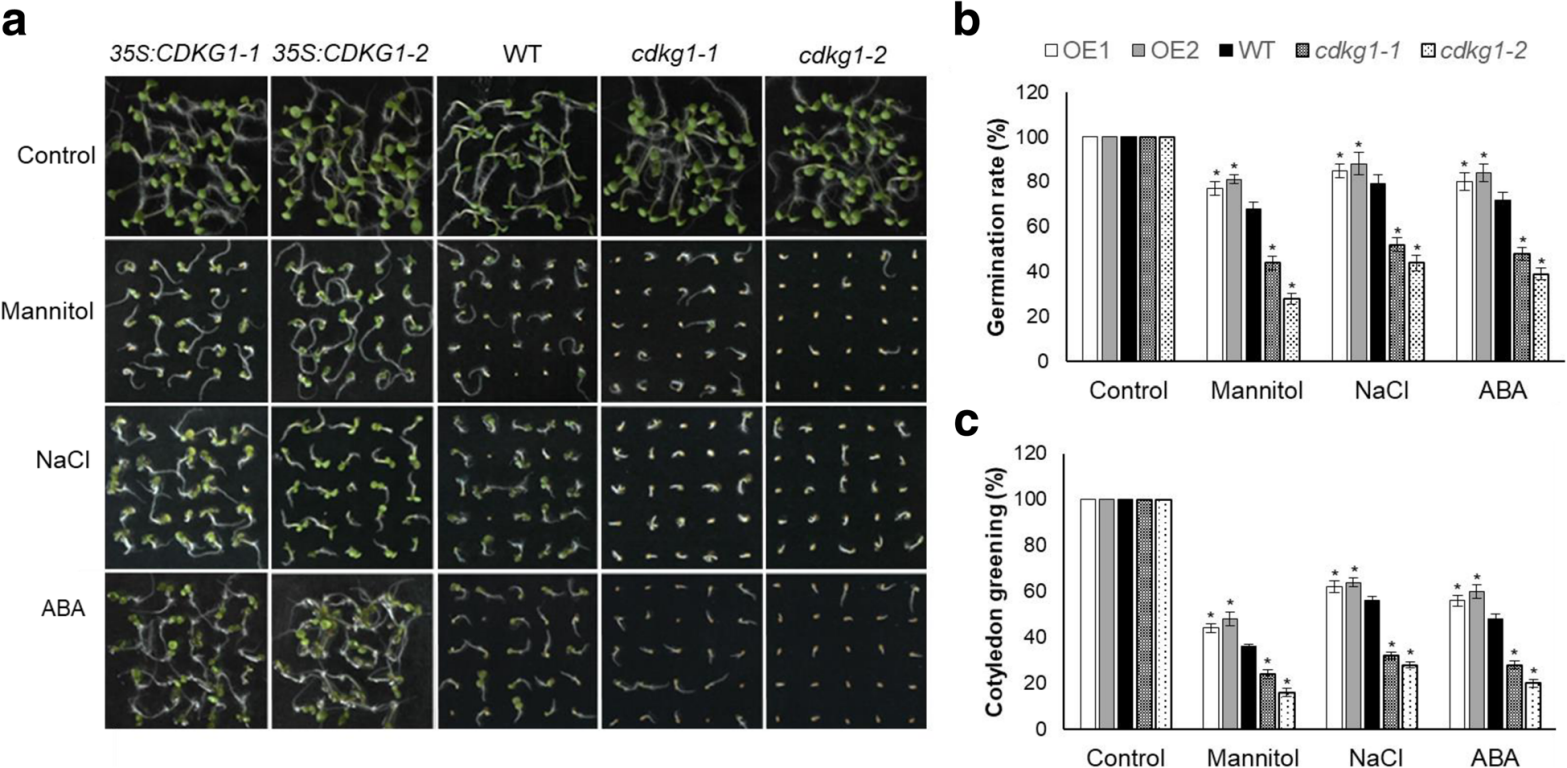
Response of seeds with altered *CDKG1* expression to ABA and abiotic stress. **a** Growth of *35S:CDKG1*, WT, and *cdkg1* seeds sown on growth media containing 300 mM mannitol, 150 mM NaCl, or 0.3 μM ABA. **b** Quantitative analysis of germination on 300 mM mannitol, 150 mM NaCl, or 0.3 μM ABA. The percentage of germinated seeds was scored 4 days after stratification. Data are means ± SD from three replicated experiments (*n* = 60). **c** Effects of osmotic stress on post-germination development of seedlings. Data were collected 7 days after germination. Each point represents a mean ± SD from three replicated experiments (n-60). Asterisks indicate a significant difference from WT by student’s *t*-test (*P* < 0.01)

Since *CDKG1* expression is induced by ABA and abiotic stress, we anticipated that seedlings of the *CDKG1* overexpressing and knockout lines could have altered responses to ABA and abiotic stress treatments. Under normal conditions, nearly 100% of the seeds from *35S:CDKG1*, WT, and *cdkg1* knockout lines germinated within 4 days after stratification (Fig. 6a). However, differences were observed in both germination rate and post-germinative development when these lines were grown on MS medium containing 150 mM NaCl. Seeds of both *35S:CDKG1* lines germinated earlier than WT seeds, while germination of *cdkg1* mutant seeds was delayed (Fig. 6a). By 4 d after stratification, the germination rates of *35S::CDKG1–*1 and *35S::CDKG1–*2 lines were 85 and 88%, respectively, while 79% of WT seeds germinated and only 53% of *cdkg1–1* and 44% for *cdkg1–2* seeds germinated under the same conditions (Fig. 6b). Consistent with germination, the proportion of seedlings with green cotyledons under salt stress 7 days after stratification remained somewhat higher for *35S::CDKG1* lines than for WT seedlings, while only about 32% of *cdkg1* seedlings were able to green their cotyledons (Fig. 6c).

To investigate whether the stress protection associated with CDKG1 overexpression is specific to salt treatments or is a more general response to osmotic stress, *35S:CDKG1* and *cdkg1* seeds were germinated and grown on MS medium containing 300 mM mannitol. Germination and cotyledon greening was severely inhibited in *cdkg1* lines under this osmotic challenge with only 45% of *cdkg1–1* and 28% of *cdkg1–2* seeds germinated by 4 days after stratification and 24 and 16%, respectively, showed green cotyledons by 7 days post-stratification (Fig. 6b). In contrast, an average of 78% of the *35S:CDKG1* seeds germinated and about 65% of these seedlings had expanded green cotyledons. Under these conditions, 68% of WT seeds germinated and 36% had green cotyledons. These results indicate that, like SINA2, CDKG1 promotes germination and early seedling development during osmotic stress.

Since plant responses to osmotic stress are regulated, at least in part, by ABA, we also examined the response of these seeds to ABA treatment. Compared with WT, *35S:CDKG1* seeds were more ABA tolerant, while seeds of *cdkg1* mutants were more ABA sensitive (Fig. 6b). After 4 days in the presence of 0.3 μM ABA, only 48% of *cdkg1–1* and 39% of *cdkg1–2* seeds germinated, but 72% of WT seeds and 80% of *35S:CDKG1–1* and *84% of 35S:CDKG1–2* seeds germinated. Likewise, after 10 days, only 28% of *cdkg1–1* and 20% of *cdkg1–2* seedlings had green cotyledons but 48% of WT, 56% of *35S:CDKG1–1* and 60% of *35S:CDKG1–2* plants had green cotyledons (Fig. 6b, c). Seeds from an additional, previously characterized *cdkg1*^*null*^ T-DNA knock-out line (SALK_075762; [5, 16, 49]), were also evaluated for their tolerance to mannitol, NaCl, and ABA treatments and reductions in germination rate similar to those with seeds from *cdkg1–1* and *cdkg1–2* mutant lines were seen (see Additional file 1: Figure 5). These results indicate that, like SINA2, CDKG1 reduces seed and seedling responsiveness to ABA under osmotic stress.

### SINA2 is phosphorylated by CDKG1 to activate its function

**Fig. 7:**
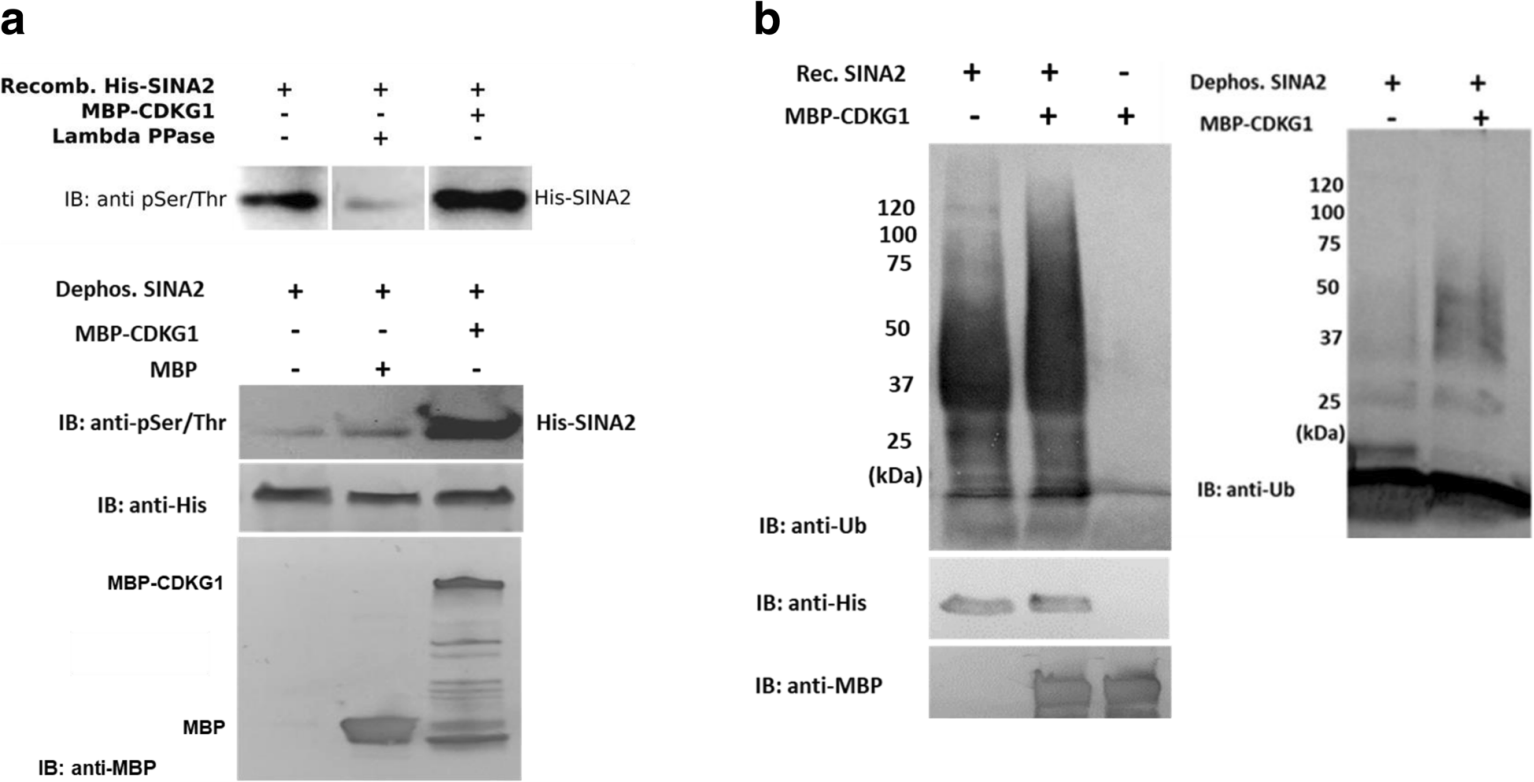
CDKG1 phosphorylates SINA2 and regulates its E3 ubiquitin ligase activity. **a** Recombinant SINA2 reacts strongly with anti phos Ser/Thr antibody in immunoblot assays. Reactivity was strongly reduced after in vitro dephosphorylation of recombinant SINA2 with λ protein phosphatase and increased after treatment, in vitro, with MBP-CDKG1. Dephosphorylated SINA2 was phosphorylated at Ser/Thr sites by MBP-CDKG1 but not by MBP in vitro. Immunoblots were probed with anti-His and anti–MBP as loading controls. **b** Increased E3 ubiquitin ligase activity of SINA2 phosphorylated by CDKG1 is indicated by enhanced accumulation of high molecular weight ubiquitinated forms in auto-ubiquitination assays. Dephosphorylated SINA2 shows reduced auto-ubiquitination activity that can be rescued by CDKG1-dependent phosphorylation

Phosphorylation is a post-translational mechanism used to modulate the activity of some E3 ubiquitin ligases [41, 46], including the human Seven in absentia homolog SiaH2 [21]. Therefore, we examined whether SINA2 is phosphorylated by CDKG1 and whether this putative phosphorylation affects its ubiquitin ligase activity. Surprisingly, recombinant His-SINA2 fusion protein isolated from *E. coli* showed substantial immunoreactivity with antibodies directed against phosphoserine and phosphothreonine (pSer/Thr) in immunoblot assays (Fig. 7a); thus, this tagged protein appears to be phosphorylated in *E. coli.* To confirm this observation, purified His-SINA2 fusion protein was treated with λ protein phosphatase to remove the putative *E. coli*-mediated phosphorylation. As predicted, dephosphorylated His-SINA2 showed reduced immunoreactivity with anti pSer/Thr (Fig. 7a) and incubation of this de-phosphorylated SINA2 with MBP-CDKG1 and ATP resulted in strongly increased anti-pSer/Thr immunoreactivity in immunoblot assays. These results show that CDKG1 can catalyze the Ser/Thr phosphorylation of dephosphorylated SINA2 in vitro. An anti-MBP immunoblot was used as a loading control, which showed that the MBP-CDKG1 fusion protein sample included minor bands, which could be truncated protein fragments or breakdown products that run toward the bottom of the gel.

To determine the effect of CDKG1-mediated Ser/Thr phosphorylation on SINA2 ubiquitin ligase activity, auto-ubiquitination assays were performed with SINA2 under various phosphorylation states (Fig. 7b). Relative to ubiquitination assays with native recombinant His-SINA2 (from *E. coli*), assays containing His-SINA2 that had been phosphorylated with MBP-CDKG1 showed an increase in high molecular weight poly-ubiquitin chains that ran near the origin of the polyacrylamide gel. Moreover, HIS-SINA2 that was dephosphorylated by treatment with λ protein phosphatase showed very weak auto-ubiquitination activity, while CDKG1-dependent re-phosphorylation of this dephosphorylated His-SINA2 rescued its activity to a considerable extent. We interpret these results to indicate that CDKG1 is able to phosphorylate SINA2 in vitro and the level of phosphorylation affects its ubiquitin ligase activity. As shown in Additional file 1: Figure S6, CDKG1 is not ubiquitinated by SINA2 under the assays conditions used. Thus, the hypothesis that CDKG1 may serve as a substrate in in vitro ubiquitination assays can be ruled out.

## Discussion

Seven in absentia-like proteins represent a clade of the Tumor Necrosis factor Receptor-Associated Factor (TRAF) protein family. These proteins are characterized by a C-terminal coiled-coil domain (known as the TRAF domain), which is required for homo- or heterodimerization and interaction with their cognate receptors or cytoplasmic signaling proteins (Xie et al., 2013). Like TRAF1, SINA2 lacks a conserved RING domain, and is predicted to lack ubiquitin ligase activity yet it retains biological activity [1]. Protein sequence alignment among SINAs from Arabidopsis showed that, while SINA2 lacks most of the N-terminal sequences found in other SINAs, it retains a modified B-box2-like zinc finger domain. The B-box2 domain of the microtubule-associated E3 ubiquitin ligase MID1 adopts a conserved ββα RING-like motif that coordinates two bound zinc ions with the same cross-brace pattern of B-box1 and RING domains. Additionally, the B-box2 domain of TRIM29, which does not contain a RING domain, also adopts a ββα RING-like fold with two bound zinc ions. Furthermore, the B-box2 domains of MID2, MURF1/TRIM63, and TRIM39 confirm the conservation of this fold between the B-box2 domain and the RING domain, suggesting that these domains may have evolved from a common ancestor and may have similar functions [23]. Our results indicate that, contrary to expectation, recombinant SINA2 retains auto-ubiquitin ligase activity in vitro. This finding lead us to speculate that the B-box2-like domain of SINA2 may be able to act as a substitute for the absent RING domain. Clearly, however, much more extensive structural and functional analysis of the B-box2 domain of SINA2, including assays of the ubiquitin ligase activity of mutagenized SINA2 forms in which the B-box2 domain has been specifically disrupted, will be needed to determine if this motif is required for the ubiquitin ligase activity of SINA2.

The phytohormone ABA plays a vital role in plant acclimation to environmental stress by retarding seed germination and cotyledon greening [30, 44, 50]. In growing plants, ABA accumulation in response to drought or salt stress conditions causes stomatal closure to reduce water loss and induces the expression of stress-protective genes [39]. Accumulating evidence shows that E3 ubiquitin ligases play essential roles in ABA signaling pathways. For example, SDIR1, a RING finger E3 ubiquitin ligase in Arabidopsis, positively regulates stress-responsive ABA signaling [48], while the RING type E3 ubiquitin ligase CaAIR1 plays a negative role in regulating ABA sensitivity and drought tolerance in pepper [33]. Two A20 and An1 domain-containing stress-associated proteins (AtSAP5 and AtSAP9), which were shown to have ubiquitin ligase activity, positively affected ABA responsiveness in Arabidopsis seedlings and mature plants [18, 19] while the related OsiSAP7 from rice negatively regulated ABA and water-deficit stress signaling [38]. ABA-dependent stomatal closure and reactive oxygen (ROS) signaling in response to drought stress are mediated by the Arabidopsis E3 ubiquitin ligase AtATL78 [43].

Previous work showed that loss of function *sina2* mutants had reduced tolerance to water deficit, while increased SINA2 expression in transgenic plants was associated with an ABA-dependent improvement in drought tolerance mediated by stomatal closure and decreasing water loss [1]. While Bao et al. [1] concentrated primarily on the effects of altered SINA2 expression on drought stress response in mature plants rather than seedlings, this group did show data for the effects of ABA on germination and seedling growth in a supplemental figure. However, they were unable to detect significant differences between WT and *sina2* mutants or *SINA2* overexpressing plants. In contrast, our results showed moderate but significant differences in seed germination and seedling growth between WT and *sina2* mutants in response to ABA treatment and more substantial differences in response to salt or osmotic stress. Thus, our data differ substantially from that of Bao et al. [1] and could indicate that the SINA2-dependent amelioration of stress-induced growth inhibition of seedling development could be mediated, at least in part, by ABA independent mechanisms.

SINA2 was found to physically interact with CDKG1 in yeast two-hybrid screening and this interaction was confirmed in vitro by pull-down assays and *in planta* by BiFC imaging. Our results also indicate that CDKG1 is involved in the regulation of stress tolerance during seed germination and cotyledon maturation. Overexpression of CDKG1 resulted in higher rates of germination and enhanced cotyledon greening, while the loss-of-function mutants were more sensitive to ABA and abiotic stress. Based on these results it seems possible that CDKG1 could act upstream of SINA2 in the regulation of ABA-dependent and/or ABA-independent stress responses. Since our results indicate that CDKG1 can affect SINA2 ubiquitin ligase activity (Fig. 7b), it seems likely that the effect of CDKG1 on ABA signaling is indirect.

The interaction between the Ser/Thr protein kinase CDKG1 and SINA2 which, as show in Fig. 3, has E3 ubiquitin ligase activity, could have at least two possible regulatory outcomes. First, SINA2 could ubiquitinate CDKG1 and target it for degradation by 26S proteasomes. Alternatively, CDKG1 could phosphorylate SINA2, possibly affecting its ubiquitin ligase activity. While these outcomes are not mutually exclusive, our results provide evidence for the second of these alternatives.

Strong immunoreactivity was detected between His-SINA2 and the pSer/Thr phosphorylation-specific antibody. This reactivity was greatly reduced when the His-SINA2 protein was treated with λ protein phosphatase and it became stronger following treatment with recombinant MBP-CDKG1. Furthermore, the loss of anti pSer/Thr immunoreactivity of recombinant His-SINA2 protein that had been treated with λ protein phosphatase was recovered after treatment with MBP-CDKG1. Though surprising, we believe that the most parsimonious interpretation of these results is that His-SINA2 is phosphorylated in *E. coli* and can be phosphorylated in vitro by CDKG1. While it is generally believed that recombinant proteins from bacterial hosts are not phosphorylated, it is clear that bacteria have Ser/Thr protein kinases that could phosphorylate recombinant proteins ([7, 12, 28]). Since recombinant His-SINA2 has ubiquitin ligase activity and this activity appeared to be enhanced by treatment with MBP-CDKG1 (Fig. 7b), we asked whether phosphorylation of His-SINA2 was required for its ubiquitin ligase activity. As shown in the right side image of Fig. 7b, the ubiquitin ligase activity of dephosphorylated His-SINA2 was greatly reduced but could be restored by treatment with MBP-CDKG1. It is unlikely that this apparent increase in ubiquitin ligase activity is due to the use of MBP-CDKG1 as a substrate since numerous attempts to detect the specific ubiquitination of MBP-CDKG1 by His-SINA2 failed (see, for example, Additional file 1: Figure S6).

Phosphorylation by protein kinases has been shown to regulate the activity of several E3 ubiquitin ligases. For example, PINK1-dependent Ser65 phosphorylation of Parkin results in sequential unfolding that enables its E3 ubiquitin ligase activity by allowing an N-terminal ubiquitin-like domain to bind to ubiquitin-loaded E2 [4]. The catalytic activity of the E3 ubiquitin ligase CHYR1 in Arabidopsis depends on phosphorylation at Thr-178 in the RING domain by SnRK2.6. This modification promotes ABA and drought responses in plants, while overexpression of a non-phosphorylatable CHYR-T178A mutant interfered with the ubiquitin ligase activity of native CHYR1 [8]. CDKs are also involved in phosphorylation of E3 ubiquitin ligases. For example, the anaphase-promoting complex/cyclosome (APC/C), a multisubunit E3 ubiquitin ligase that promotes cell cycle progression and genome stability, is activated by phosphorylation by CDK1 [9]. Our results show that SINA2 is phosphorylated by CDKG1 in vitro, which appears to activate its E3 ubiquitin ligase activity. Based on the protein structure of SINA2 and the consensus phosphorylation motif (RXXS/T) recognized by Ser/Thr kinases [2, 26], we tentatively identify Ser88 and Thr161 as potential sites in SINA2 that could be phosphorylated by CDKG1. Future experiments using mutant SINA2 forms with substitutions at these sites will be used to test our hypothesis that CDKG1-dependent phosphorylation of SINA2 is required for its E3 ubiquitin ligase activity.

## Conclusions

Although it lacks a canonical RING domain, our data show that SINA2 has E3 ubiquitin ligase activity. It is possible that a modified B-box domain may substitute for the RING domain but further experiments are required to test this hypothesis. Moreover, SINA2 physically interacts with CDKG1, which appears to modulate its ubiquitin ligase activity through phosphorylation. *CDKG1* expression is up-regulated by ABA and osmotic stress and it plays a role in maintaining seed germination and early seedling development during abiotic stress.

## Methods

### Plant materials and growth conditions

The *Arabidopsis thaliana* ecotype Col-0 was used as the WT in this study. Seeds of *sina2* T-DNA insertion mutant lines (*sina2–3*: Salk_129594 and *sina2–1*: Salk_099866) and *cdkg1* (*cdkg1–1*: Salk_066975 and *cdkg1–2*: Salk_096690) were obtained from the Arabidopsis Biological Resource Center (ABRC). Seeds were surface-sterilized with 75% ethanol for 1 min, followed by 30% chlorine bleach for 10 min and extensively washed five times with sterile water. Sterilized seeds were plated on ½-strength MS plates supplemented with or without specified concentrations of ABA, NaCl, or mannitol. The plated seeds were stratified for 3 days at 4 °C and then transferred to a growth chamber at 22 °C and 70% relative humidity under a 16 h-light/8 h-dark photoperiod. Screening the homozygous T-DNA insertion lines was validated by PCR analysis with gene-specific primers listed in Additional file 1: Table S1. Germination (defined as the protrusion of the radicle through the seed coat) and cotyledon greening and expansion were scored every day after transfer to the growth chamber, and cotyledon greening and expansion rates were calculated over the total of germinated seeds. Young vegetative *Nicotiana benthamiana* plants (1 to 1.5-month-old) were used as host plants for agroinfiltration experiments.

### RNA extraction and quantitative PCR analyses

Two-week-old Arabidopsis seedlings were collected and immediately frozen in liquid nitrogen. Total RNA was isolated using the Total RNA I solation Kit (Qiagen) and treated with RNase-free DNase I (Qiagen). For real-time PCR assays, 1 μg of total RNA was used for first strand cDNA synthesis using oligo (dT) primers and Iscript reverse transcriptase (Bio-Rad) according to the manufacturer’s protocol. Quantitative expression assays were carried out with iTag SYBR Green Supermix (Bio-Rad) and an ABI PRISM7000 Sequence Detection System (Applied Biosystems). Arabidopsis *ACTIN2* was used as a reference to normalize the expression of target genes. Each experiment was repeated three times with three technical repeats for each.

### Transgenic vector construction and plant transformation

Full-length *AtSINA2* cDNA (At3g13672) was amplified from a stock obtained from ABRC (U10197) using forward primer SINA2F and reverse primer SINA2R (see Additional file 1: Table S1). The PCR product was cloned into the Gateway entry vector pENTR-D-TOPO (Invitrogen) following the manufacturer’s instructions. The resulting plasmids were transformed into competent DH5α cells (Invitrogen) by heat-shock and transformants were selected with 50 μg/ml kanamycin. Full-length *CDKG1* cDNA (AT5G63370) cloned in Gateway vector pENTR-SD-TOPO was obtained from TAIR (U1643). The pENTR-D-TOPO-*SINA2* and pENTR-SD-TOPO- *CDKG1* were cut with MluI and the DNA fragments containing *SINA2* and *CDKG1* coding sequence, flanked by attL recombination sites, were recombined into the plant expression vectors pEarlyGate 101 and pGWB2 respectively. The identity of the cloned cDNAs was confirmed by sequencing. To construct the *CDKG1* overexpression vector, the full length cDNA of *CDKG1* was inserted into the pGWB Gateway plant transformation vector, in which expression of *CDKG1* is under the control of the CaMV 35S promoter [27]. The constructed vector was introduced into *Agrobacterium tumefaciens* strain GV3101 and transformed into Col-0 by floral infiltration. Transgenic plants were selected on ½-strength MS medium containing 50 mg/L kanamycin. For the phenotypic analysis, two independent T3 homozygous lines were used.

### Yeast two-hybrid assays

Yeast two-hybrid screening was performed by Hybrigenics (http://www.hybrigenics.com) S.A., Paris, France. Briefly, the full-length *SINA2* coding sequence was PCR-amplified and cloned into pB27 as a C-terminal fusion to LexA DNA-binding domain (N-LexA-SINT2-C). The construct was checked by sequencing the entire insert and used as a bait to screen a random-primed *A. thaliana* seedling cDNA library. Approximately sixty three million clones were screened using a mating approach and selected on a medium lacking tryptophan, leucine and histidine. The putative interactors were amplified by PCR and sequenced. The GenBank database (NCBI) was used to establish the identity of the interacting candidates. To confirm the interaction, full-length *CDKG1* cDNA was ligated into pGBKT7 DNA-BD and pGADT7 DNA-AD vectors using the NcoI and PstI sites to generate the bait and prey plasmids, respectively, and transformed into AH109 yeast cells containing *SINAT2* either as bait or prey plasmids. The yeast cells were plated onto SD/^−^Trp/^−^Leu medium and replica plated on SD/^−^Trp/^−^Leu/^−^His/^−^Ade medium to confirm the interaction. Plasmid pGBKT7 and pGADT7 were used as negative controls.

### Protein expression and pull-down assays

The *SINA2* open reading frame (663 bp) was cloned into the pET-59-DEST vector via the Gateway system (Invitrogen) to generate pET-SINA2 containing a 6 × His-SINA2 fusion construct. The PCR product of *CDKG1* was inserted into *Xba*I and *Pst*I sites in a pMAL-c2x vector (New England Biolabs) as pMAL-CDKG1 to produce the N-terminal MBP fusion protein. These recombinant vectors were expressed in *E. coli* strain BL21 (DE3) by induction overnight with 0.5 mM isopropyl β-D-1-thiogalactopyranoside (IPTG) at 16 °C. Recombinant 6 × His-SINA2 fusion protein was purified by using Ni agarose beads (GE Healthcare), and MBP-CDKG1 fusion protein was purified according to the pMAL protein fusion and purification system (E8200, NEB). Approximately 5 μg of MBP-AtCDKG1 fusion protein was immobilized on amylose beads and incubated with His-tagged SINA2 proteins. After incubation at 4 °C on a rotary incubator for 2 h, the beads were washed five times with ice-cold buffer (20 mM Tris-HCl, 200 mM NaCl, 1 mM EDTA, 1 mM DTT). The MBP protein was used as a control in the assay. Bound proteins were re-suspended by boiling in SDS gel-loading buffer for 5 min and separated by SDS-PAGE. After running the gel, protein bound to MBP-AtCDKG1 was detected by Western blotting with anti-His antibody.

### Localization and bimolecular fluorescence complementation (BiFC) assay

For subcellular localization of CDKG1, the coding sequences of AtCDKG1 was introduced into the pGWB406 Gateway plant transformation vector. For BiFC analysis, *SINA2* cDNA was cloned into pSITE-DEST-nEYFP-C1 vector and fused with the N-terminal fragment of YFP to form YFPN-SINA2 construct. The AtCDKG1 cDNA was cloned into pSITE-DEST-cEYFP-C1 vector and fused with C-terminal fragment of YFP to generate YFPC-AtCDKG1 construct. The resultant constructs were transformed into the *Agrobacterium tumefaciens* GV2260 strain. For transient expression, *Agrobacterium* transformed with the appropriate binary plasmids was infiltrated into *N. benthamiana* leaves individually for subcellular localization or in pairs for BiFC assay. Leaf samples were collected 2 days after agroinfiltration and GFP or YFP fluorescence signal was observed by a laser scanning confocal microscope (Leica DM IRE2) at the Samuel Roberts Noble Foundation Imaging Core Facility.

### In vitro ubiquitination assays

Ubiquitination assays were carried out by adding 5 μg ubiquitin (Boston Biochem), 150 nM human E1 (Boston Biochem), 200 nM human recombinant UbcH2 (Boston Biochem), and 5 μg purified His-SINA2 in a reaction buffer containing 50 mM Tris, pH 7.5, 2 mM ATP, 5 mM MgCl2, 2 mM DTT. The reaction mixture (30 μl) was incubated at 30 °C for 2 h and stopped by heating to 100 °C for 5 min in SDS-PAGE sample buffer (125 mM Tris-HCl, pH 6.8, 20% glycerol, 4% SDS, and 10% β-mercaptoethanol). Protein samples were separated by 10% SDS-PAGE and immunoblots were probed using anti-Ubi antibody and Anti-Mouse IgG secondary antibody produced in goat (Sigma). Bands were visualized using chemiluminescence as instructed by the manufacturer (Amersham Pharmacia).

### In vitro de-phosphorylation and phosphorylation assays

The purified His-SINA2 fusion protein was dialyzed against a 1000-fold volume buffer containing 20 mM MOPS (pH 7.5) and 1 mM DTT. For de-phosphorylation assay, His-SINA2 fusion protein was incubated with λ protein phosphatase (NEB) for 2 h at 30 °C. For phosphorylation, His-SINA2 and purified MBP-CDKG1 were incubated together in a final volume of 40 μl in kinase buffer (50 mM HEPES-KOH, PH 7.9, 10 mM MnCl_2_, 1 mM DTT and 0.2 mM ATP) at 30 °C for 1 h. For immunoblot analysis, the reaction was terminated by adding SDS-loading buffer and boiling for 5 min.

## Additional file

Additional file 1: **Figure S1.** Amino acid sequence alignment between Arabidopsis SINAT1 (NP_181729.1), SINAT2 (NP_191363.1), SINAT3 (NP_567118.1), SINAT4 (NP_194517.1), SINAT5 (AAM11573) and SINA2. * indicates conserved amino acid moieties of the RING domain, ↓ indicates conserved amino acids of the putative B-box2 domain. Identical amino acid regions are shaded black and conserved sequences are shown in gray. **Figure S2.** (a) Reciprocal yeast two hybrid assay and (b) Yeast two hybrid deletion analysis of SINA2. Coding sequences were recombined into pDEST-AD and pDEST-BD, transformed into AH109 yeast cells and plated onto SD Trp^−^Leu^−^ medium and replica plated on SD Trp^−^Leu/^−^His^−^Ade^−^ medium. **Figure S3.** (a) Comparison of the PK domains from *Arabidopsis thaliana* (CDKG1, NP_201142 and CDKG2, NP_176925), *A. lyrata* (AlCDKG1, XP_002866550), *Camelina sativa* (CsCDKG1, XP_010484064), *Brassica rapa* (BrCDKG1, XP_009130249), *B. oleracea var. oleracea* (BoCDKG1, XP_013628692) and *Taraenaya hassleriana* (ThCDKG1, XP_010545957). Identical sequences are shown in black and areas conserved sequences are shaded gray. (b) Phylogenetic tree of PK domains of CDKG protein sequences was generated using the neighbor-joining method with MEGA. Bootstrap values (> 50%) from 500 replicates are shown. **Figure S4.** Sub-cellular localization of GFP-CDKG1 and GFP CFKG1. *Agrobacterium* strains carrying *35S:GFP*, *35S:GFP-SINA2*, and *35S:GFP-CDKG1* were infiltrated into *Nicotiana benthamiana* leaves, and the GFP signals from leaves that transiently express GFP-CDKG1 or GFP alone, as indicated, were analyzed by confocal microscopy. **Figure S5.** Germination rate of SALK_075762 * cdkg1* seeds in response to treatment with 200 mM mannitol, 200 mM NaCl, or 100 μM ABA was scored 4 days after stratification. Data represent means of 60 seeds ± SD. Asterisks indicate significant differences from WT (*P* < 0.01). **Figure S6.** Failure of SINA2 to ubiquitinate CDKG1. Ubiquitination of CDKG1 by SINA2 was assayed in the presence of human E1, human E2 and ubiquitin and samples were resolved by SDS-PAGE and detected using anti-MBP. **Table S1.** Primers used in this study. (PDF 1326 kb)

## Abbreviations

ABA: Abscisic acid
BiFC: Bimolecular fluorescence complementation
CDK: Cyclin-Dependent kinase
GFP: Green Fluorescent protein
HECT: Homologous to the E6-AP Carboxyl Terminus
MS: Murashige and Skoog
RING: Really Interesting New Gene
SINA: Seven in absentia
UPS: Ubiquitin/26S proteasome system
YFP: Yellow fluorescent protein
