# Supplementary material for: Phosphorylation of Arabidopsis SINA2 by CDKG1 affects its ubiquitin ligase activity
Source: BMC Plant Biol. 2018 Jul 16;18:147. doi: 10.1186/s12870-018-1364-8 (PMC6048857; doi:10.1186/s12870-018-1364-8)
Supplement: Supplementary file 1 — Figure S1. Amino acid sequence alignment between Arabidopsis SINAT1 (NP_181729.1), SINAT2 (NP_191363.1), SINAT3 (NP_567118.1), SINAT4 (NP_194517.1), SINAT5 (AAM11573) and SINA2. * indicates conserved amino acid moieties of the RING domain, ↓ indicates conserved amino acids of the putative B-box2 domain. Identical amino acid regions are shaded black and conserved sequences are shown in gray. Figure S2. (a) Reciprocal yeast two hybrid assay and (b) Yeast two hybrid deletion analysis of SINA2. Coding sequences were recombined into pDEST-AD and pDEST-BD, transformed into AH109 yeast cells and plated onto SD Trp−Leu− medium and replica plated on SD Trp−Leu/−His−Ade− medium. Figure S3. (a) Comparison of the PK domains from Arabidopsis thaliana (CDKG1, NP_201142 and CDKG2, NP_176925), A. lyrata (AlCDKG1, XP_002866550), Camelina sativa (CsCDKG1, XP_010484064), Brassica rapa (BrCDKG1, XP_009130249), B. oleracea var. oleracea (BoCDKG1, XP_013628692) and Taraenaya hassleriana (ThCDKG1, XP_010545957). Identical sequences are shown in black and areas conserved sequences are shaded gray. (b) Phylogenetic tree of PK domains of CDKG protein sequences was generated using the neighbor-joining method with MEGA. Bootstrap values (> 50%) from 500 replicates are shown. Figure S4. Sub-cellular localization of GFP-CDKG1 and GFP CFKG1. Agrobacterium strains carrying 35S:GFP, 35S:GFP-SINA2, and 35S:GFP-CDKG1 were infiltrated into Nicotiana benthamiana leaves, and the GFP signals from leaves that transiently express GFP-CDKG1 or GFP alone, as indicated, were analyzed by confocal microscopy. Figure S5. Germination rate of SALK_075762 cdkg1 seeds in response to treatment with 200 mM mannitol, 200 mM NaCl, or 100 μM ABA was scored 4 days after stratification. Data represent means of 60 seeds ± SD. Asterisks indicate significant differences from WT (P < 0.01). Figure S6. Failure of SINA2 to ubiquitinate CDKG1. Ubiquitination of CDKG1 by SINA2 was assayed in the presence of human E1, human E2 and [file 12870_2018_1364_MOESM1_ESM.pdf]

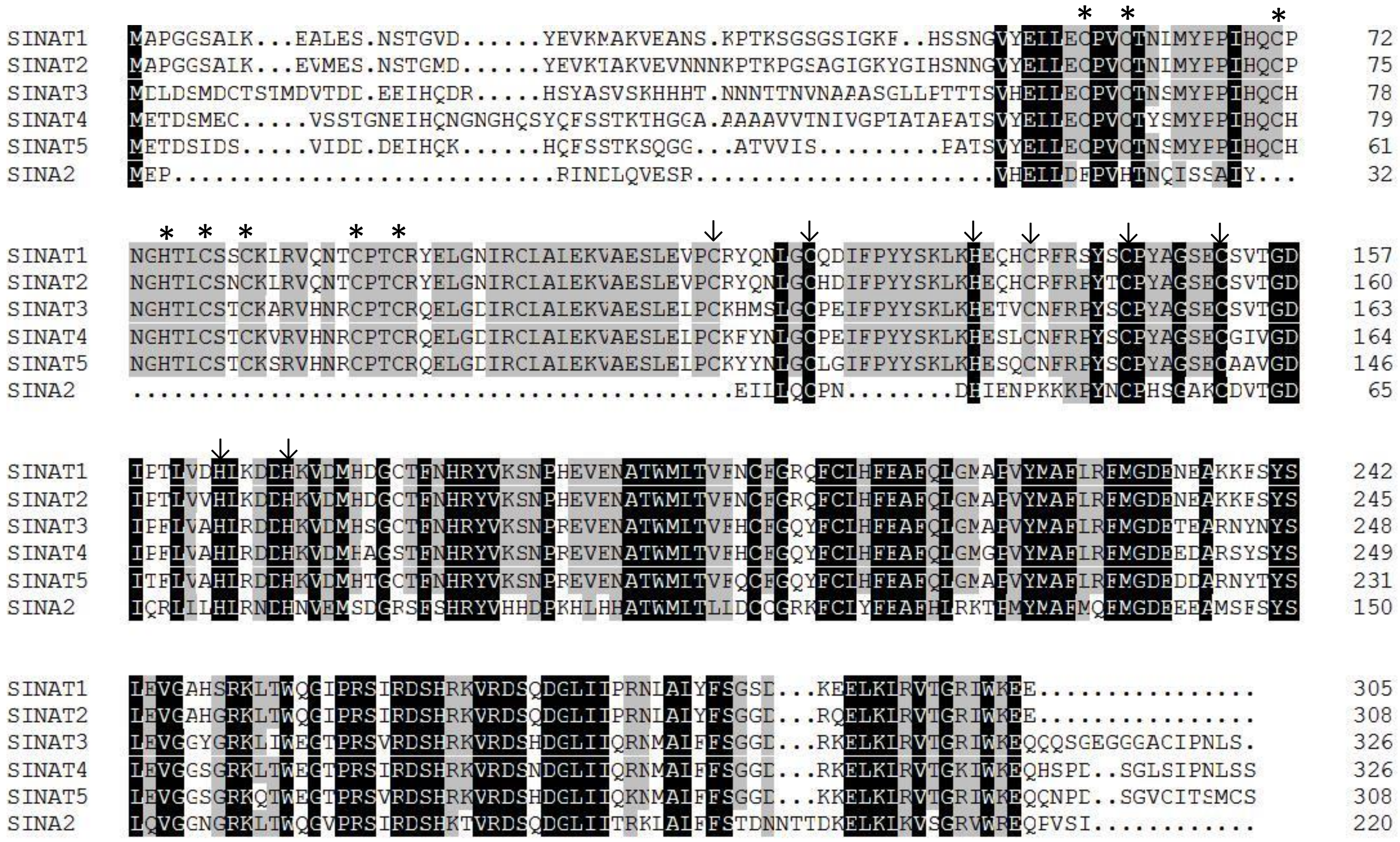

Supplementary Figure 1. Amino acid sequence alignment between SINA2 and other Arabidopsis SINAs. Comparison of Arabidopsis SINAT1 (NP_181729.1), SINAT2 (NP_191363.1), SINAT3 (NP_567118.1), SINAT4 (NP_194517.1), SINAT5 (AAM11573) and SINA2. * indicates conserved amino acids of the RING domain, ↓ indicates conserved amino acids of the putative B-box2 domain.

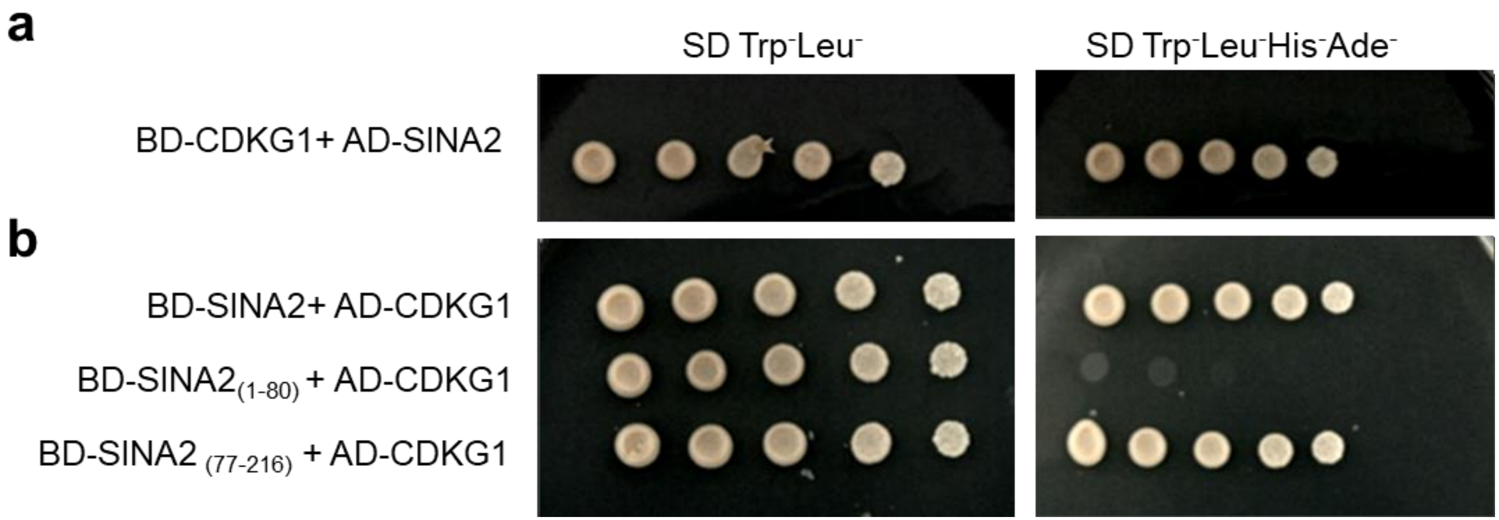

Supplementary Figure 2. Interaction between SINA2 and CDKG1. (a) Reciprocal yeast two hybrid assay. (b) Yeast two hybrid deletion analysis of SINA2. the cDNA's and PCR fragments were initially cloned into pENTR-D-TOPO (Invitrogen) and recombined in pDEST-AD and pDEST-BD and transformed into AH109 yeast cells. The yeast cells were plated onto SD $Trp^{-}Leu^{-}$ medium and replica plated onto SD $Trp^{-}Leu^{-}His^{-}Ade^{-}$ medium.

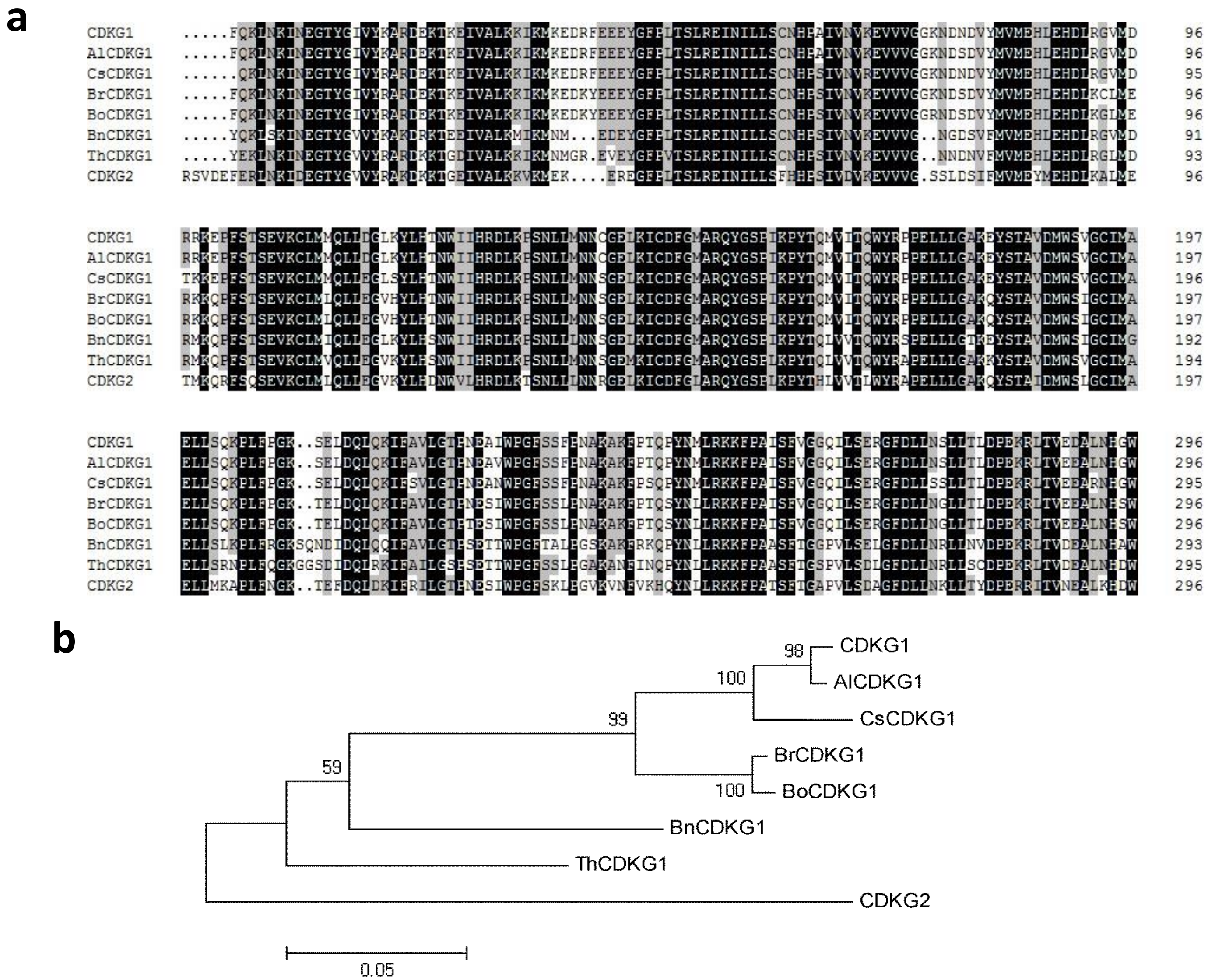

Supplementary Figure 3. Alignment and phylogenic tree of PK domain amino acid sequences of CDKGs. (a) Comparison of the PK domains from *Arabidopsis thaliana* (CDKG1, NP_201142 and CDKG2, NP_176925), *Arabidopsis lyrata* (AlCDKG1, XP_002866550), *Camelina sativa* (CsCDKG1, XP_010484064), *Brassica rapa* (BrCDKG1, XP_009130249), *Brassica oleracea var. oleracea* (BoCDKG1, XP_013628692) and *Taraenaya hassleriana* (ThCDKG1, XP_010545957). Identical amino-acid sequences in all eight sequences are shown in black. (b) A phylogenetic tree of PK domains of different CDKG proteins. Alignment was constructed by cluster W and the neighbor-joining method was used to generate the phylogenetic tree using the MEGA program. The numbers indicate the bootstrap values (>50%) from 500 replicates. The scale indicates branch lengths.

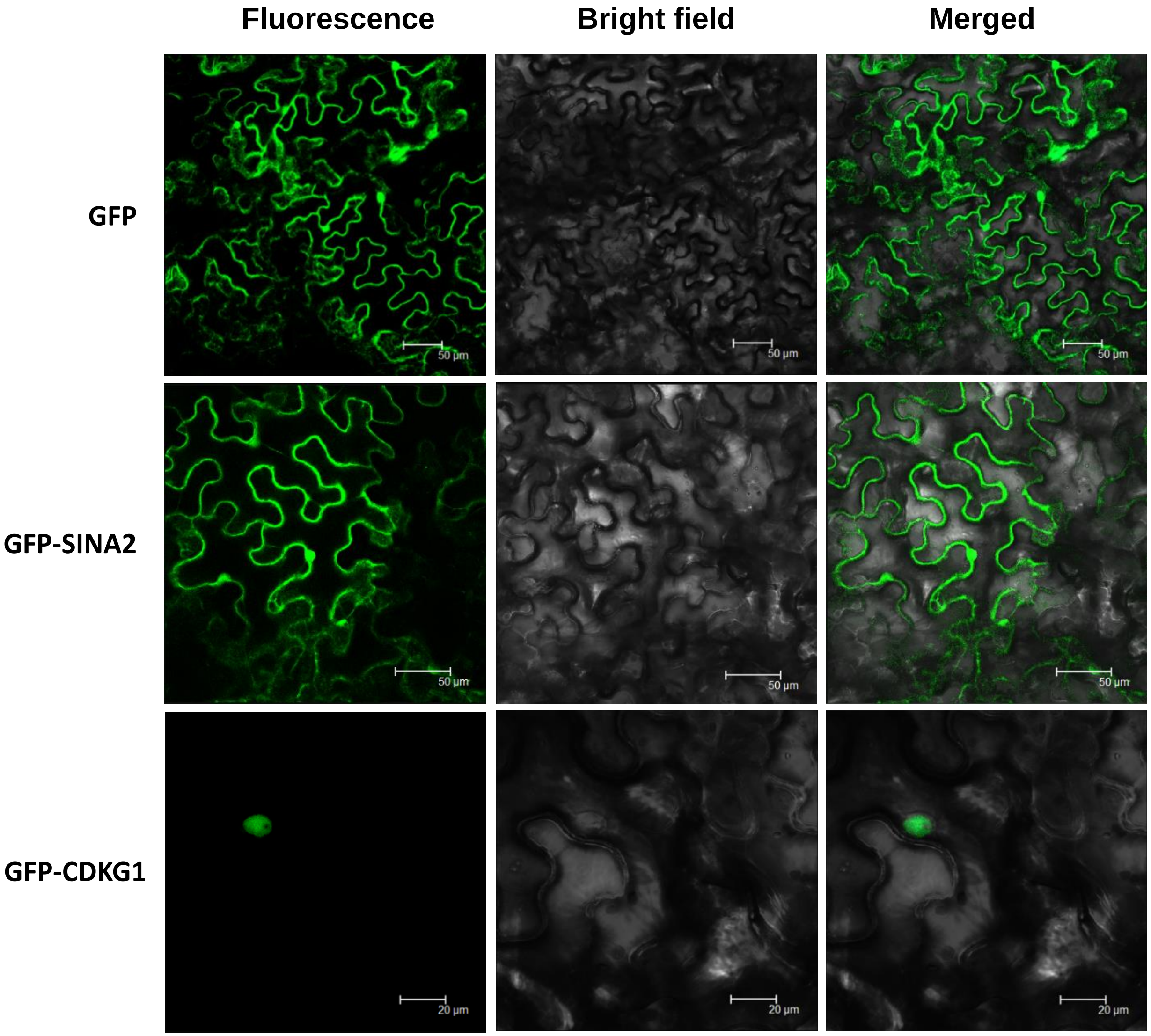

Supplementary Figure 4. Sub-cellular localization of the GFP-CDKG1 protein. The *Agrobacterium* strains carrying the plasmids *35S:GFP, 35S:GFP-SINA2, and 35S:GFP-CDKG1* were infiltrated into *Nicotiana benthamiana* leaves, and the GFP signals from leaves that transiently express GFP-SINA2, GFP-CDKG1, or GFP alone, as indicated, were analyzed by confocal microscopy 48 h after agroinfiltration.

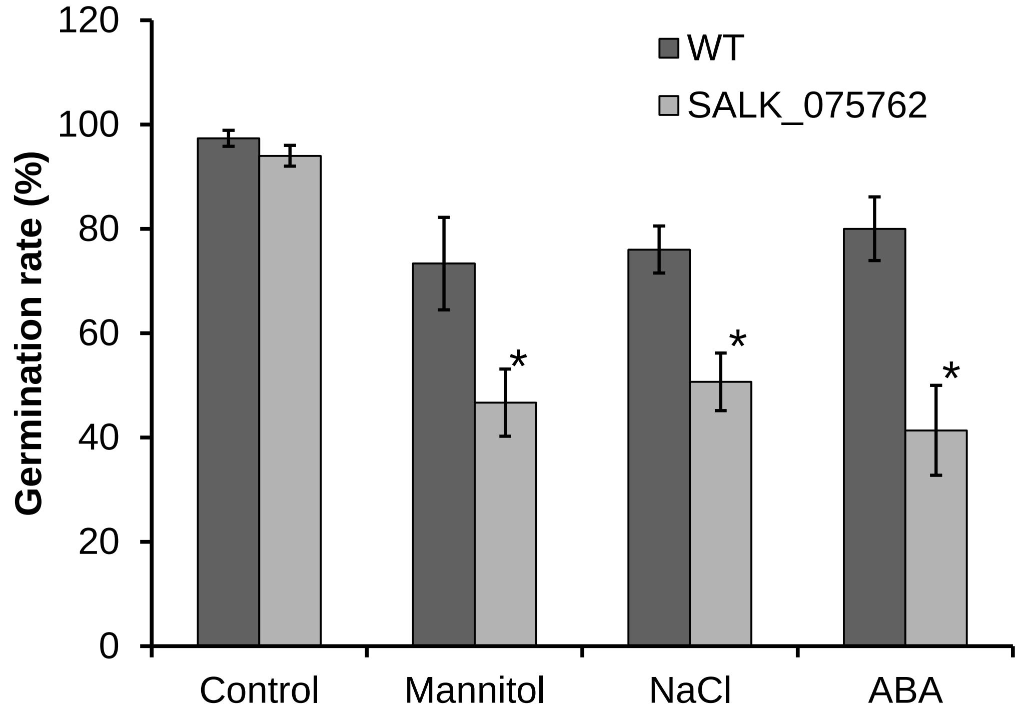

Supplementary Figure 5. Germination rate of SALK_075762 *cdkg1* seeds in response to treatment with 200 mM mannitol, 200 mM NaCl, or 100 µM ABA. The percentage of germinated seeds was scored 4 days after stratification. Each data point represents a mean of 60 seeds ± SD. Asterisks indicate significant differences from WT by student's *t*-test (P<0.01).

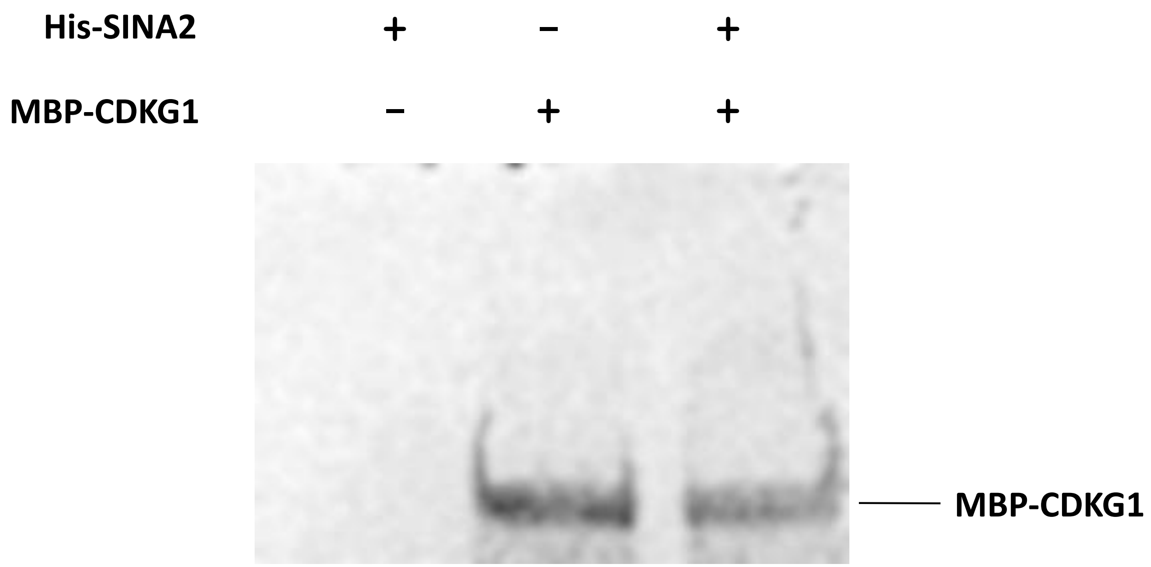

Supplementary Figure 6 . Failure of SINA2 to ubiquitinate CDKG1. Assays to detect the ubiquitination of CDKG1 by SINA2 was performed in the presence of human E1, human E2 (UbcH5a) and ubiquitin at 30°C for 2 h. Samples were resolved on a 7.5% SDS-PAGE gel and detected by immunoblot analysis using anti-MBP No ubiquitinated MBP-CDGK1 was detected in multiple assays.

Supplementary Table 1. Primers used in this study.

**Primer name**	**Primer sequence**
sina2-LP1	5’TATCCAAGTGAAGGCATCCAC3’
sina2-RP1	5’AGGAGCCAAGTGTGATGTCAC3’
sina2-LP2	5’AGGAGCCAAGTGTGATGTCAC3’
sina2-RP2	5’TATCCAAGTGAAGGCATCCAC3’
cdkg1-LP1	5’CTTTGTTGCCTGGAGATTCAG3’
cdkg1-RP1	5’ATCTGATGGTGTCAGAGTCGG3’
cdkg1-LP2	5’ATTGGGTTGGGATGGTTATTG3’
cdkg1-RP2	5’ATCTGATGGTGTCAGAGTCGG3’
LBa1	5’TGGTTCACGTAGTGGGCCATCG3’
SINA2F	5’CACCATGGAACCTCGAATCAATG3’
SINA2R	5’TCATATCGAAACAGGCTGTTC3’
RT-SINA2F	5’TCAGGTGGAATCTCGGGTT3’
RT-SINA2R	5’ATGTGGTCGTTGGGACACT3’
RT-qPCR-CDKG1F	5’ GCCTTACACCCAGATGGTTATT3’
RT-qPCR-CDKG1R	5’ CAACCCACTGACCACATATCA3’
ACTIN2F	5’GGAAGGATCTGTACGGTAAC3’
ACTIN2R	5’GGACCTGCCTCATCATACT3’
SINA2(1-80)-F	5'caccATGGAACCTCGAATCAATG3'
SINA2(1-80)-R	5'TCACCCATCACTCATTTCAACATTGTG3'
SINA2(77-216)-F	5'caccATGAGTGATGGGCGTAGTTTC3'
SINA2-77-216-R	5'TCATATCGAAACAGGCTGTTCTC3'
